# Drug-Susceptible and Multidrug-Resistant *Mycobacterium tuberculosis* in a Single Patient 

**DOI:** 10.3201/eid2511.180638

**Published:** 2019-11

**Authors:** Anthony Baffoe-Bonnie, Eric R. Houpt, Lauren Turner, Denise Dodge, Scott K. Heysell

**Affiliations:** Virginia Polytechnic Institute and State University Carilion School of Medicine, Roanoke, Virginia, USA (A. Baffoe-Bonnie);; University of Virginia, Charlottesville, Virginia, USA (E.R. Houpt, S.K. Heysell);; Division of Consolidated Laboratory Services, Richmond, Virginia, USA (L. Turner);; Virginia Department of Health, Richmond (D. Dodge)

**Keywords:** heteroresistance, whole genome sequencing, spoligotyping, *Mycobacterium tuberculosis*, bacteria, drug resistance, tuberculosis and other mycobacteria

## Abstract

A patient who had initial infection with mixed strains of drug-susceptible and multidrug-resistant tuberculosis was presumed to have acquired drug resistance before confirmation that sequential strains were genotypically distinct. Transmitted infection with mixed strains is likely underappreciated; identifying these infections requires spoligotyping and whole-genome sequencing.

Multidrug-resistant (MDR) tuberculosis (TB), defined as infection with *Mycobacterium tuberculosis* that is resistant to isoniazid and rifampin, can be transmitted and manifest as a primary infection without a patient having received those medications or can be acquired by the patient during drug therapy. A person may be initially infected with >1 *M. tuberculosis* strain with different patterns of drug resistance (*1**–**4*). We present such a case, which is likely uncommon but underappreciated; identification requires spoligotyping and whole-genome sequencing of sequential strains.

A 28-year-old man came to a hospital in Virginia, 1 year after immigrating from the Philippines, with a 4-week history of fevers, night sweats, weight loss, voice change, and cervical lymphadenopathy. A computed tomography scan showed no lung parenchymal or pleural abnormality. Cervical lymph node biopsy showed caseating granulomas with acid-fast bacilli (AFB). Sputum smears demonstrated 4+ AFB. Rapid nucleic acid amplification testing by commercial line probe assay confirmed *M. tuberculosis* complex from both sites without *rpoB*, *katG*, or *inhA* mutation. Test results for HIV were positive; HIV viral load was 521,800 copies/mL and CD4 count 7 cells/mm^3^. Diagnoses were made of TB lymphadenitis and presumed laryngeal TB. We started the patient on antiretroviral drugs after initiating isoniazid, rifampin, ethambutol, and pyrazinamide. Phenotypic testing ultimately showed susceptibility to isoniazid, rifampin, ethambutol, and pyrazinamide. The patient achieved sputum culture conversion to negative before beginning his eighth week of treatment and transitioned to a continuation phase of isoniazid and rifampin 5 days/week.

Twenty weeks into treatment, the patient’s sputum AFB culture obtained 12 weeks after initiation was reported as positive. Given this relapse, we conducted therapeutic drug monitoring for isoniazid and rifampin. Estimated peak serum drug concentrations were 2.07 (range 3–5) μg/mL for isoniazid and 5.98 (range 8–24) μg/mL for rifampin. Molecular sequencing of the week 12 *M. tuberculosis* isolate at the Centers for Disease Control and Prevention (*5*) found mutations in *rpoB* (GACCAG>GAG; Asp516Gln517Glu) and *inhA* (C-15T), but the subsequently tested pretreatment sputum isolate was confirmed negative for mutations in the resistance-determining regions of *rpoB* (rifampin), *katG* and *inhA* (isoniazid), and *pncA* (pyrazinamide). Both isolates had mutations in the *embB* gene Leu355Leu (silent) and Glu378Ala (reported as unlikely to cause resistance alone and, more commonly, a marker of Indo-Oceanic strain lineage) (*6*). We switched the patient’s drug regimen to levofloxacin, linezolid, capreomycin, para-aminosalicylate, ethambutol, and pyrazinamide. Phenotypic susceptibility tests later confirmed MDR TB: 25% resistance to isoniazid at 1.0 μg/mL and 100% resistance to rifampin at 1.0 μg/mL. The patient continued with this MDR TB regimen for 15 months after culture conversion to negative. He has remained healthy. 

We clinically interpreted this scenario as one of acquired drug resistance, likely contributed by the subtarget concentration of anti-TB drugs. However, analyses of mycobacterial interspersed repetitive unit–variable-number tandem-repeat typing, along with insertion sequence 6110 restriction fragment length polymorphism analyses of the pretreatment drug-susceptible strain and the week 12 MDR strain, showed distinct spoligotypes. Whole-genome sequencing differed by >100 single nucleotide polymorphisms, supporting that these strains were genotypically distinct. Upon further questioning, the patient related that before immigrating he lived in a small apartment in Manila where friends and family would frequently lodge before seeking treatment at the city’s referral hospital.

Prior treatment with isoniazid and rifampin is a major risk factor for MDR TB. Comprehensive epidemiologic studies and improved access to whole-genome sequencing have revealed that primary MDR TB transmitted to the patient can be common (*7*). Our case adds to the literature on transmitted MDR TB dynamics and shows how initial infections with mixed strains may be an underreported cause of treatment failure. The clonal diversity from a *M. tuberculosis* sample is clearly attenuated following conventional culture techniques on solid agar (*8*). Although heteroresistance at drug-resistant loci of *M. tuberculosis* can be detected at low levels with newer next-generation sequencing techniques from cultured growth, these analyses cannot distinguish infection with multiple strains from heteroresistance among subpopulations of the same strain (*9*).

As with the previous cases (*1**–**4*) of mixed-strain infection, the patient we report did not have prior history of TB treatment and showed initial improvement but then had a recrudescence of symptoms and culture reversion 115 days into first-line therapy (range 90–150 days in previously reported cases) (Table). Applying whole-genome sequencing to additional samples collected from our patient and meticulous isolation of different colonies of cultured growth might have detected heteroresistance, and consequent alteration of the initial treatment regimen could have prevented the recrudescence of disease. However, the detection of lower-level genetic heteroresistance has not been rigorously studied for its effect on populations of persons initiating TB treatment and will undoubtedly vary based on the drug-specific locus that is heteroresistant and the quantitative level of detection. Well-curated prospective cohorts that contribute sequencing data may refine our understanding of this effect but will require nuanced bioinformatics *(**10**)*.

**Table:** Characteristics of cases of treatment failure resulting from concurrent infection with mixed strains of drug-resistant Mycobacterium tuberculosis*

Patient age, y/sex	Immune status	Site(s) of infection	Time to recurrence	Platform for strain identification	Patient country of origin	Country of diagnosis	Reference
24/M	Immunocompetent	Lymph node, gastric aspirate	100 d	Mixed-linker fingerprint PCR	Nepal	Germany	(*1*)
23/M	HIV negative	Pulmonary	150 d	Spoligotyping and MIRU	Somalia	USA	(*4*)
24/M	HIV negative	Pulmonary, gastric aspirate	90 d	IS6110 RFLP and spoligotyping	Kazakhstan	Germany	(*2*)
62/M	HIV negative	Pulmonary	120 d	MIRU-VNTR	Portugal	UK	(*3*)
28/M	HIV positive	Lymph node, trachea	84 d	Spoligotyping and WGS	Philippines	USA	This study

*IS, insertion sequence; MIRU-VNTR, mycobacterial interspersed repetitive unit–variable number tandem repeat; RFLP, restriction fragment length polymorphism; WGS, whole genome sequencing.

## References

[1] Theisen A, Reichel C, Rüsch-Gerdes S, Haas WH, Rockstroh JK, Spengler U, et al. Mixed-strain infection with a drug-sensitive and multidrug-resistant strain of *Mycobacterium tuberculosis.* Lancet. 1995;345:1512–3. 10.1016/S0140-6736(95)91073-5 7769926

[2] Niemann S, Richter E, Rüsch-Gerdes S, Schlaak M, Greinert U. Double infection with a resistant and a multidrug-resistant strain of *Mycobacterium tuberculosis.* Emerg Infect Dis. 2000;6:548–51. 10.3201/eid0605.000518 10998389 PMC2627962

[3] Hingley-Wilson SM, Casey R, Connell D, Bremang S, Evans JT, Hawkey PM, et al. Undetected multidrug-resistant tuberculosis amplified by first-line therapy in mixed infection. Emerg Infect Dis. 2013;19:1138–41. 10.3201/eid1907.130313 23764343 PMC3713993

[4] Mendez MP, Landon ME, McCloud MK, Davidson P, Christensen PJ. Co-infection with pansensitive and multidrug-resistant strains of *Mycobacterium tuberculosis.* Emerg Infect Dis. 2009;15:578–80. 10.3201/eid1504.080592 19331736 PMC2671451

[5] Yakrus MA, Driscoll J, Lentz AJ, Sikes D, Hartline D, Metchock B, et al. Concordance between molecular and phenotypic testing of *Mycobacterium tuberculosis* complex isolates for resistance to rifampin and isoniazid in the United States. J Clin Microbiol. 2014;52:1932–7. 10.1128/JCM.00417-14 24648563 PMC4042757

[6] Campbell PJ, Morlock GP, Sikes RD, Dalton TL, Metchock B, Starks AM, et al. Molecular detection of mutations associated with first- and second-line drug resistance compared with conventional drug susceptibility testing of *Mycobacterium tuberculosis.* Antimicrob Agents Chemother. 2011;55:2032–41. 10.1128/AAC.01550-10 21300839 PMC3088277

[7] Shah NS, Auld SC, Brust JCM, Mathema B, Ismail N, Moodley P, et al. Transmission of extensively drug-resistant tuberculosis in South Africa. N Engl J Med. 2017;376:243–53. 10.1056/NEJMoa1604544 28099825 PMC5330208

[8] Martín A, Herranz M, Ruiz Serrano MJ, Bouza E, García de Viedma D. The clonal composition of *Mycobacterium tuberculosis* in clinical specimens could be modified by culture. Tuberculosis (Edinb). 2010;90:201–7. 10.1016/j.tube.2010.03.012 20435520

[9] Operario DJ, Koeppel AF, Turner SD, Bao Y, Pholwat S, Banu S, et al. Prevalence and extent of heteroresistance by next generation sequencing of multidrug-resistant tuberculosis. PLoS One. 2017;12:e0176522. 10.1371/journal.pone.0176522 28545050 PMC5436647

[10] McNerney R, Clark TG, Campino S, Rodrigues C, Dolinger D, Smith L, et al. Removing the bottleneck in whole genome sequencing of *Mycobacterium tuberculosis* for rapid drug resistance analysis: a call to action. Int J Infect Dis. 2017;56:130–5. 10.1016/j.ijid.2016.11.422 27986491

